# Occupational exposure to organic solvents and the risk of developing testicular germ cell tumors (TESTIS study): Effect of combined exposure assessment on risk estimation

**DOI:** 10.5271/sjweh.4161

**Published:** 2024-07-01

**Authors:** Margot Guth, Corinne Pilorget, Marie Lefevre, Astrid Coste, Aurélie Danjou, Brigitte Dananché, Delphine Praud, Olivia Pérol, Myriam Daudin, Marie-Ange Clarotti, Stéphanie Lattes, Céline Bouillon, Adèle Paul, Joachim Schüz, Louis Bujan, Ann Olsson, Béatrice Fervers, Barbara Charbotel, the TESTIS study group

**Affiliations:** 1 UMRESTTE, UMR T 9405, IFSTTAR, Lyon 1 University, Lyon University, Eiffel University, Lyon, France.; 2 Santé publique France - French National Public Health Agency, Saint-Maurice, France.; 3 INSERM UMR1296 Radiation: Defense, Health, Environment, Lyon, France.; 4 Prevention Cancer Environnement Departement, Centre Léon Bérard, Lyon, France.; 5 Inserm, Epidemiology of Childhood and Adolescent Cancer (CRESS UMR1153 / EPICEA) - Université Paris Cité, France.; 6 Environment and Lifestyle Epidemiology Branch, International Agency for Research on Cancer/World Health Organization (IARC/WHO), 25 avenue Tony Garnier, CS 90627, 69366 LYON, Cedex 07, France.; 7 Fédération Française des CECOS, Paris, France.; 8 Université de Toulouse; UPS; Groupe de Recherche en Fertilité Humaine (EA 3694, Human Fertility Research Group), CECOS, Toulouse, France.; 9 Biologie de la reproduction, CHU Caen Normandie, Caen, France.; 10 Pôle de biologie-pathologie, laboratoire de biologie de la reproduction-Cecos, CHU de Nice, hôpital Archet-II, Professeur Michiels, université de Nice-Sophia-Antipolis, Nice, France.; 11 Service de médecine et Biologie de la Reproduction, CHRU de Tours, F-37044, Tours, France.; 12 DEFE (Développement Embryonnaire, Fertilité, Environnement) INSERM 1202 Universités Montpellier et Toulouse 3, CECOS Hôpital Paule de Viguier, CHU de Toulouse, Toulouse, France.; 13 CRPPE-Lyon, Hospices Civils de Lyon, Lyon, France.

**Keywords:** exposure assessment method, occupational health, risk assessment, testicular cancer, trichloroethylene

## Abstract

**Objectives:**

Etiological factors of testicular germ cell tumors (TGCT) remain largely unknown, but a causal role of occupational exposures to solvents has been suggested. Previous studies analyzing these exposures reported discordant results, potentially related to exposure assessment methods. The aim of this study was to investigate the role of occupational exposure to solvents on the risk of developing TGCT among young men.

**Methods:**

This study examined occupational exposures to solvents and TGCT risk based on the lifetime work histories of 454 cases and 670 controls, aged 18–45 years, of the French national TESTIS case–control study. Solvent exposure was estimated using: (i) exposure assignment by job-exposure matrix (JEM) and (ii) JEM combined with self-reported exposure data from specific questionnaires (SQ) and expert assessment (EA). Odds ratios (OR) and 95% confidence intervals (CI) were estimated using conditional logistic regression models.

**Results:**

Both approaches (JEM and JEM+SQ+EA) showed a consistent association between TGCT and trichloroethylene exposure (exposed versus not exposed; JEM=OR 1.80 [95% confidence interval (CI) 1.12–2.90] and JEM+SQ+EA= OR 2.59 (95% CI 1.42–4.72). Both approaches also observed positive associations with ketone esters and fuels & petroleum-based solvents.

**Conclusion:**

The results suggest that some organic solvents might be involved in the pathogenesis of TGCT among occupationally exposed men. The combined use of JEM+SQ+EA seemed to limit misclassification by considering individual exposure variability and is, therefore, an appealing approach to assess occupational exposures in epidemiological studies.

With over 74 000 new cases worldwide in 2020, testicular cancer ranks as the 20^th^ most common malignancy, but it is the most frequent cancer among Caucasian males aged 15–44 years (1). Malignant testicular germ cell tumors (TGCT) are the prevailing testicular cancers (>95%) (1), and are thought to develop from germ cell neoplasia *in-situ* (GCNIS-TGCT), which are presumed to derive from a defect in the normal maturation of fetal germ cells from primordial germ cells *in utero* (2). Several studies have also advanced the hypothesis of a two-stage development, combining this initial intra-uterine alteration followed by a malignant transformation later in life such as early adulthood (3, 4).

It is recognized that the pathogenesis of testicular cancer has a strong environmental component, but its exogenous risk factors are not well known (5). Most studies of testicular cancer associated with environmental and occupational exposures have been limited to studies examining specific occupations (6). Recently, the International Agency for Research on Cancer (IARC) classified firefighter occupational exposure as “carcinogenic to humans”, with limited evidence for testicular cancers (7). Previously (8), we observed increased TGCT risk among agricultural, electrical and electronics workers, suggesting a possible role of exposure to solvents. Workers use solvents in many industries, and several are known or suspected carcinogens (9). Few epidemiological studies have examined the link between occupational exposure to solvents and TGCT risk, and available findings are inconsistent (10).

Retrospective exposure assessment is challenging but essential to detect relevant exposure–disease associations (11, 12). Various methods may be used for occupational exposure assessment in case–control studies such as job-exposure matrices (JEM), self-assessment via specific questionnaires (SQ), and expert assessment (EA) (13). Each of these commonly used exposure assessment methods have limitations. The major limitation of JEM is that individuals with the same job title are assigned the same exposure not accounting for individual variation in exposures within the same job (12). Self-reported data may provide useful information for solvent exposure (14, 15) yet, over- or underestimation of risk due to differential recall bias is a concern when assigning exposure based on these data. Therefore, the use of hybrid approaches, combining several retrospective assessment methods, has been suggested to improve the exposure characterization to the substances of interest (12, 16, 17). This study aimed to assess the association between occupational exposure to selected solvents [alcohols, ketones and esters (KetEst), trichloroethylene (TCE), perchloroethylene (PCE), methylene chloride (MC) and fuels & petroleum-based (F&P) solvents] and TGCT risk in a French national case–control study using two exposure assessment approaches (JEM and JEM+SQ+EA).

## Methods

### Study design and population

TESTIS is a French multi-center case–control study on TGCT risk factors. Participants aged 18–45 years were recruited from 20 university hospital centers in metropolitan France. The study design and data collection have been described in detail earlier (8, 18).

Briefly, cases of histologically confirmed TGCT referred to research centers for the preservation of eggs and sperm (*Centres d’étude et de conservation des oeufs et du sperme, CECOS*) for sperm cryopreservation prior to treatment were identified between January 2015 and April 2018. A medical oncologist performed case ascertainment and reviewed the pathology reports and serum tumors markers. Cases were classified as seminoma or non-seminoma, according to the International Classification of Disease for Oncology (ICD-O) and the World Health Organization classification of tumors of the urinary system and male genital organs (19). Testicular cancers not originating from GCNIS (ie, spermatocytic, epidermoïd cysts, neuroendocrine, Leydig cell, hemangiomas and Sertoli cell tumors) were excluded. As the proportion of false-positive TGCT was low (5.1%), TGCT cases with missing pathology report (N=43, 7.8%) were included as cases (18). Two control groups with no personal history of testicular cancer or cryptorchidism were recruited. The group A controls were sperm donors and partners of women consulting for fertility disorders, both with normal sperm production, attending the CECOS. The group B controls were partners of women managed for a pathological pregnancy in specialized maternity clinics, adjacent to the CECOS. As the individual matching initially planned (one case for one control A and one control B, matched on year of birth (+/-3 years) and hospital center’s region) was not possible for all participants, we performed frequency-matching on year of birth grouped into 5-year categories, and region of recruitment (8).

Overall, 1463 subjects were eligible and invited to participate in the study. Of these, 1367 agreed to participate (supplementary material, www.sjweh.fi/article/4161, figure S1). Further, 96 cases and 147 controls were excluded for various reasons (eg, non-GCNIS TGCT, missing date of diagnosis or incomplete interviews, etc.) leaving 1124 participants (454 cases, 384 group A and 286 group B controls) for these analyses.

### Data collection

A trained interviewer (IPSOS Company) blinded to the participants’ case–control status, administered a general questionnaire and collected personal information, including detailed lifetime work history. For each job period, information on job title, industry title, tasks performed, and year of start and end of the job was recorded. The general questionnaire included filter questions (eg, “Have you used or been exposed by or via co-workers to solvents, thinners, or degreasing products?”) for specific exposures.

If a participant reported potential exposure to solvents in one of the filter questions in a given job, a SQ was administered collecting information on exposure to alcohols, KetEst, TCE, PCE, MC and F&P solvents (including white spirit, kerosene, gasoline fuels - among others), as well as the context of use and frequency of their use (supplementary table S1).

We only considered exposure occurring before the index date, defined for cases as the date of TGCT diagnosis, and for controls as the date at which controls within each stratum were of the same age as the median age of diagnosis of cases in this stratum.

### Exposure assessment

The first approach consisted of applying the Matgéné JEM. An industrial hygienist coded each job period according to the International Standard Classification of Occupation 1968 version (ISCO-68) and the French classification of economic activities [1999 version, updated version of NAF 1993 (NAF-99)]. Occupational exposure to alcohols, KetEst, TCE, PCE, MC, and ≥1 F&P solvents (benzene; automobile gasoline; white spirits and other aromatics; diesel, kerosene and fuel oil; special petroleum products and other aliphatics) were assessed by JEM (one for each solvent or solvent group) developed for the French general population in the context of the Matgéné program (20). The solvent exposure categories presented in this study are those for which an assessment was available both via Matgéné’s JEM and via self-reported solvent exposures (SQ).

For each job (combination of ISCO and NAF), three indices of exposure were provided by the JEM: probability of exposure (P) expressed as the percentage of workers exposed in this job; intensity of exposure (I) expressed as the average atmospheric concentration to which the worker is exposed during direct and indirect exposure tasks; and frequency of exposure (F) expressed as a percentage of working time with solvent exposure. For exposure to ≥1 F&P solvents, the JEM provided the P and an average level of exposure (L) during a usual working day, combining I and F indices. Exposure indices were available for different periods depending on the solvent: 1947–2021 for F&P solvents; 1950–2021 for alcohols, KetEst, TCE, PCE, and MC. Solvent exposure levels have considerably evolved over the last 60 years, due to changes in raw materials, techniques, working conditions and regulations. As a result, each of the JEM has been historicized, ie each job has been broken down into as many periods as necessary, in order to take these changes into account (20). For example, for TCE exposure, for the same job, indices (P, I, F) varied over four periods: 1950–1969; 1970–1994; 1995–2012; 2013–2021 (supplementary table S2). The P, I and F indices are defined in semi-quantitative or qualitative classes (eg, not exposed, low-, medium-, high-exposed) in the Matgéné JEM (20). Supplementary table S3 discloses the classes of the different exposure indices for each solvent and solvent group and the weighting assigned to each by the industrial hygienists who developed the JEM.

**Figure 1:**
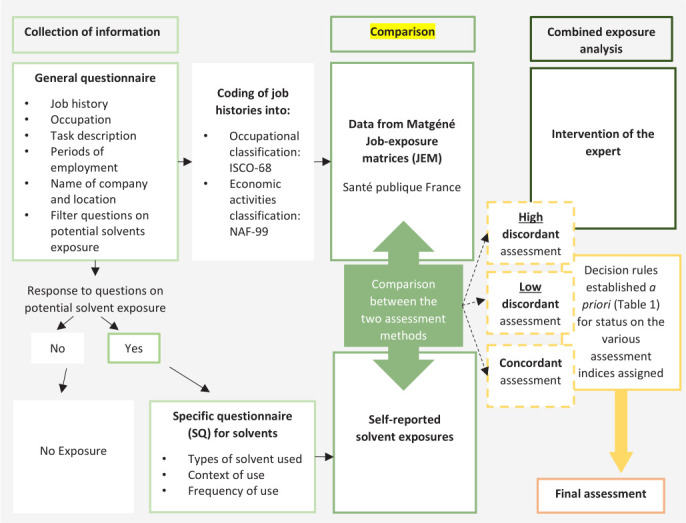
Pathways of exposure assessment to selected organic solvents by job exposure matrices (JEM) and self-reported exposures in specific questionnaires (SQ) in the TESTIS study. Adapted from de Ahrens et al (21).

The second approach to estimate exposure to the selected solvents consisted of using a combined exposure assessment approach (JEM+SQ+EA), involving several steps (figure 1). The first was to compare the agreement between JEM- and SQ-based exposure data for all jobs to identify jobs periods with concordant or discordant exposure assessment between the two methods. Sensitivities, specificities, and positive (PPV) and negative predictive values (NPV) were estimated, using JEM assessment as “gold-standard”. As a measure of concordance, the Kappa statistics were calculated.

To reduce exposure misclassification, an industrial hygienist individually reviewed jobs with discordant exposure assessments, ie, assessed by the JEM as exposed [or not exposed] while assessed by the SQ as unexposed [or as exposed]. Based on the subject’s statements (job titles, periods of employment, tasks performed, context and frequency of solvent use), the expert classified the job as exposed/unexposed.

Finally, based on comparison of JEM- and SQ-based assessment data and with the intervention of an EA, decision rules were established *a priori* for status on the various assessment indices assigned (table 1). This decision algorithm allowed us to obtain a single assessment (including P, I and F) for a job-period.

**Table 1:** Combined approach: Decision algorithm for exposure assessment combining job-exposure matrix (JEM), specific questionnaires (SQ) and expert assessment (EA). [P=probability ; I=intensity ; F=frequency.]

Condition number	Concordance ^a^	JEM assessment	SQ assessment	EA assessment	Final status	Probability	Level
1	1	Exposed	Exposed	-	Exposed	100%	JEM
2	1	Not exposed	Not exposed	-	Not exposed	0	0
3	0	Exposed (P≥70%)	Not exposed	Not exposed	Not exposed	0	0
				Exposed	Exposed	JEM	JEM
4	0	Exposed (10% ≤P<70%)	Not exposed	-	Exposed	JEM	JEM
5	0	Exposed (P<10%)	Not exposed	-	Not exposed	0	0
6	0	Not exposed	Exposed (F≥0.2 hrs/days)	Not exposed	Not exposed	0	0
				Exposed	Exposed	100%	F_SQ_ × I_EA_
7	0	Not exposed	Exposed (F<0.2 hrs/days)	-	Not exposed	0	0

^a^ Concordance between JEM- and SQ-based data: 1=agreement between JEM-based and self-reported exposure assessment; 0=discordant, ie, assessed by the JEM as exposed [or not exposed] while assessed by the SQ as unexposed [or exposed].

In the combined approach (JEM+SQ+EA), for jobs that both the JEM and SQ assessed as being exposed, the P was estimated at 100% and the L was maintained as assessed by the JEM (table 1, supplementary table S4). Jobs assessed by both methods as unexposed were considered unexposed. Job periods with P of exposure estimated at ≥70% by the JEM – but as unexposed by the SQ (condition n°3) – were classified as exposed or unexposed based on the EA. If the EA estimated that this job period was exposed, the P and L assigned by the JEM were maintained. Job periods with JEM-based P of 10–70% (condition n°4) were classified according to the exposure indices assigned by the JEM. Job periods with JEM-based P of <10% (condition n°5), were classified as non-exposed. Jobs assessed as exposed by the SQ with F of ≥0.2 hours/day but estimated as unexposed by the JEM (condition n°6), were classified as exposed or unexposed based on the EA. If the EA estimated that this job period was exposed, the P was estimated to be 100%, due to the agreement between the SQ and EA. F was determined according to the SQ, and the I was set by the EA according to the knowledge available in the literature, in relation to the exposure process described by the subject in the SQ. The L was calculated as the product of the self-reported exposure frequency and the I done by the expert (F_SQ_×I_EA_). Job-periods with SQ-based F of <0.2 hours/day (condition n°7) were classified as non-exposed.

Finally, linking the JEM indices with occupational histories, the following exposure variables were obtained for each subject: “never/ever” exposed to a specific solvent, with ≥1 job with a P of >0. A job-specific exposure score (JES) was then calculated as te result of the product of P, F and I (or P×L) weights, and duration of the job in months. We weighted the JES obtained by the time worked (ie, a 70%-time job was multiplied by 0.7 etc.). Then, for each study subject, a cumulative exposure score (CES) was calculated by summing the JES over the entire work history. To examine potential effects of the highest exposures, CES were also categorized as “never exposed” (referent group) and two categories of exposed workers: “low” and “high” exposed, according to the 50^th^ percentile among exposed controls (supplementary table S5). This statistical method has been applied to the JEM and combined (JEM+SQ+EA) assessment method.

### Covariates

According to a method described in detail previously (8), TGCT risk factors identified from the literature, associated with TGCT in a univariate analysis (P<0.20), were included in a single regression model. A manual backward stepwise selection procedure was then performed to select variables significantly associated with TGCT (P<0.05): sibship size, being born from multiple pregnancy, personal history of testicular trauma, family history of TGCT, and family history of cryptorchidism.

### Statistical analysis

The distribution of characteristics of the study population was expressed as mean and standard deviation (SD) for continuous variables or number (frequency and percentage) for categorical variables. Conditional logistic regression models were used to model associations between solvents and TGCT risk, using the unexposed group of each solvent individually as the reference category. Odds-ratios (OR) and corresponding 95% confidence intervals (CI) were adjusted for covariates. Given the broad age strata (5-year categories), we further adjusted for age to avoid residual confounding by age in birth age strata.

Additionally, we conducted sensitivity analyses: we excluded cases with a personal history of cryptorchidism (N=40) and TGCT cases not confirmed by pathology reports (N=43). Finally, the analyses were repeated by classifying jobs with a P of 10–70% by the JEM only, as unexposed (condition n°4, table 1). All analysis were performed using SAS statistical software version 9.4 (SAS Institute Inc, Cary, NC, USA).

## Results

The main characteristics of cases and controls are presented in table 2. Compared to controls, cases tended to be younger [cases mean age 31.9 (SD 6.1) years; controls: 33.6 (SD 5.4) years], were more likely to be first born (P=0.04) and born from a multiple pregnancy (P=0.02). Cases were also more likely to report a history of inguinal hernia (P=0.01), testicular trauma (P=0.004), and family history of TGCT (P=<0.001) and family history of cryptorchidism (P=0.01) than controls.

**Table 2:** Characteristics of testicular germ cell tumors (TGCT) cases and controls (group A and group B), TESTIS study, 2015–2018.

Characteristics		TGCT cases (N=454)		Controls (N=670)	P-value ^a^
		N (%)		N (%)	
Age at diagnosis (cas) / inclusion (controls) (years)					< 0.001
	≤25	64 (14.1)		32 (4.8)	
	26–30	106 (23.4)		144 (21.5)	
	31–35	113 (24.9)		239 (35.7)	
	36–40	85 (18.7)		162 (24.2)	
	≥41	43 (9.5)		93 (13.9)	
	Missing	43 (9.5)		0 (0.0)	
Year of birth					< 0.001
	<1975	37 (8.2)		50 (7.5)	
	1975–1979	80 (17.6)		128 (19.1)	
	1980–1984	117 (25.8)		218 (32.5)	
	1985–1989	130 (28.6)		203 (30.3)	
	1990–1994	70 (15.4)		63 (9.4)	
	1995–1999	20 (4.4)		8 (1.2)	
Education					0.10
	Secondary	180 (39.7)		203 (30.3)	
	1–2-year university degree	97 (21.4)		148 (22.1)	
	>3 years university degree	128 (28.2)		234 (34.9)	
	Other	48 (10.6)		85 (12.7)	
	Missing	1 (0.2)		0 (0.0)	
Smoking status					0.12
	Never	205 (45.2)		314 (46.9)	
	Former	102 (22.5)		171 (25.5)	
	Current	147 (32.4)		185 (27.6)	
Birth weight (g)					0.49
	<2500	25 (5.5)		33 (4.9)	
	2500–4000	356 (78.4)		561 (83.7)	
	≥4000	46 (10.1)		58 (8.7)	
	Missing	27 (6.0)		18 (2.7)	
Gestational age (weeks)					0.16
	<37	32 (7.1)		34 (5.1)	
	≥37	415 (91.4)		629 (93.9)	
	Missing	7 (1.5)		7 (1.0)	
Born from multiple pregnancy					0.02
	No	433 (95.4)		652 (97.3)	
	Yes	21 (4.6)		17 (2.5)	
	Missing	0 (0.0)		1 (0.2)	
Birth order					0.04
	First	213 (46.9)		305 (45.5)	
	Second	163 (35.9)		224 (33.4)	
	Third	63 (13.9)		93 (13.9)	
	Fourth and more	15 (3.3)		48 (7.2)	
Sibship size					0.03
	1	25 (5.5)		59 (8.8)	
	2	192 (42.3)		254 (37.9)	
	3	153 (33.7)		206 (30.8)	
	≥4	84 (18.5)		150 (22.4)	
	Missing	0 (0.0)		1 (0.2)	
Personal history of inguinal hernia					0.01
	No	414 (91.2)		637 (95.1)	
	Yes	39 (8.6)		32 (4.8)	
	Missing	1 (0.2)		1 (0.2)	
Personnal history of testicular trauma					0.004
	No	387 (85.2)		611 (91.2)	
	Yes	67 (14.8)		59 (8.8)	
Family history of TGCT					< 0.001
	No	419 (92.3)		651 (97.2)	
	Yes	33 (7.3)		16 (2.4)	
	Missing	2 (0.4)		3 (0.4)	
Family history of cryptorchidism					0.01
	No	422 (93)		645 (96.3)	
	Yes	28 (6.2)		18 (2.7)	
	Missing	4 (0.9)		7 (1.0)	
Age at voice change (years)					0.61
	<12	16 (3.5)		26 (3.9)	
	12–16	375 (82.6)		532 (79.4)	
	>16	53 (11.7)		94 (14.0)	
	Missing	10 (2.2)		18 (2.7)	
Cannabis use at 12–17 yrs					0.30
	No	312 (68.7)		443 (66.1)	
	Yes	142 (31.3)		227 (33.9)	
Cannabis use frequency (12–17 yrs)					0.24
	Never	312 (68.72)		443 (66.1)	
	<Once / month	51 (11.23)		84 (12.5)	
	≥1 time / month	22 (4.85)		47 (7.0)	
	Once / week	37 (8.15)		39 (5.8)	
	Once / day	32 (7.05)		57 (8.5)	
Cannabis use (at 18–25 yrs)					0.68
	No	262 (57.71)		393 (58.7)	
	Yes	192 (42.29)		276 (41.2)	
	Missing	0 (0.0)		1 (0.2)	
Cannabis use frequency (18–25 yrs)					0.09
	Never	262 (57.7)		393 (58.7)	
	<Once / month	62 (13.7)		107 (16.0)	
	≥1 time / month	23 (5.1)		46 (6.9)	
	Once / week	41 (9.0)		54 (8.1)	
	Once / day	66 (14.5)		69 (10.3)	
	Missing	0 (0.0)		1 (0.2)	
Occupation of last employment ^b^ ^(^ISCO-68)					0.61
	Professional, technical & related workers (0/1)	130 (28.6)		230 (34.3)	
	Administration & managerial workers (2)	44 (9.7)		90 (13.4)	
	Clerical & related workers (3)	34 (7.5)		53 (7.9)	
	Sales workers (4)	36 (7.9)		49 (7.3)	
	Service workers (5)	39 (8.6)		52 (7.8)	
	Agricultural, animal husbandry & forestry workers, fishermen & hunters (6)	20 (4.4)		22 (3.3)	
	Production & related workers, transport equipment operators & laborers (7/8/9)	128 (28.2)		161 (24.0)	
	Military	1 (0.2)		2 (0.3)	
	Missing	22 (4.9)		11 (1.6)	
Industry of last employment ^b^ (NAF-99 code)					0.09
	Agriculture, hunting & forestry (A)	13 (2.9)		19 (2.8)	
	Fishing (B)	1 (0.2)		0 (0.0)	
	Mining & quarrying (C)	0 (0.0)		1 (0.2)	
	Manufacturing (D)	73 (16.1)		107 (16.0)	
	Electricity, gas & water supply (E)	12 (2.6)		13 (1.9)	
	Construction (F)	44 (9.7)		51 (7.6)	
	Wholesale & retail trade; repair of motor vehicles, motorcycles and personal & household goods (G)	64 (14.10)		69 (10.3)	
	Hotels & restaurants (H)	18 (4.0)		18 (2.7)	
	Transport, storage & communication (I)	21 (4.6)		50 (7.5)	
	Financial intermediation (J)	13 (2.9)		18 (2.7)	
	Real estate, renting & business activities (K)	63 (13.9)		98 (14.6)	
	Public administration & defence; compulsory social security (L)	41 (9.0)		66 (9.9)	
	Education (M)	22 (4.9)		40 (6.0)	
	Health & social work (N)	22 (4.9)		73 (10.9)	
	Other community, social & personal service activities (O)	23 (5.1)		34 (5.1)	
	Private households with employed persons (P)	1 (0.2)		0 (0.0)	
	Extra-territorial organizations & bodies (Q)	0 (0.0)		0 (0.0)	
	Missing or unknown	23 (5.1)		13 (1.9)	

^a^ P-values (cases and all controls) from bivariate conditional logistic regression models conditioned on region and birth year– except for the year of birth which was a matching factor. ^b^ Last job reported by the participant. If no job was held or insufficient information was provided, the job was in the 'missing' category.

Of the 4083 jobs reported by the 1124 participants, N=3813 jobs were considered for the analyses (N=94 jobs not considered due to insufficient detail; after censoring, N=176 jobs periods <6 months or started after the index date). The proportion of men ever exposed to any solvent was higher in cases than among controls (supplementary table S6). Overall, 50% have been exposed to ≥1 solvent during their professional career. As regards the most common solvent exposures, JEM found 39% of the subjects had been exposed to F&P solvents while JEM+SQ+EA method found a rate of 30%. These figure were respectively 24% and 28% for alcohols and 17% and 22% for KetEst. Each of the other exposures accounted for ≤10% of the participants.

### Comparison between JEM and SQ exposure tools

Comparison between JEM- and SQ-based assessments for the six solvents covered by the SQ (ie, alcohols, KetEst TCE, PCE, MC, and F&P solvents), yielded 2540 job periods assessed concordantly by the JEM and the SQ, and 1273 jobs periods (31%) with ≥1 discordant exposure assessment. Details for each of the conditions and corresponding decision rules, overall and by solvent, are presented in supplementary table S4. Compared to JEM, the sensitivity of SQ data was low (0.00–0.35 depending on the solvent) while the specificity was high (0.93–0.99 depending on the solvent). The range for PPV and NPV was 0.00–0.59 and 0.82–1.00, respectively. The highest PPV observed (0.59) was for exposure to F&P solvents. Cohen’s Kappa coefficients (κ) showed fair agreement between the JEM assessments and SQ data for F&P solvents, and KetEst (κ=0.27 for both solvent groups), poor agreement for MC, alcohol and TCE (κ=0.10, 0.12 and 0.15 respectively), and no agreement for PCE (κ=-0.01) (table 3).

**Table 3:** Cohen’s Kappa, sensitivity (SENS) and specificity (SPEC), and positive and negative predictive values (PPV/NPV) between self-reported individual exposures and JEM exposures.

	SENS ^a^	SPEC ^a^	Cohen’ Kappa	PPV ^a^	NPV ^a^
Alcohols	0.15	0.94	0.12 (0.08–0.16)	0.30	0.87
Ketones and esters	0.35	0.93	0.27 (0.22– 0.32)	0.32	0.94
Petroleum solvents	0.28	0.95	0.27 (0.24–0.31)	0.59	0.82
Trichloroethylene	0.16	0.97	0.15 (0.08–0.21)	0.19	0.97
Perchloroethylene	0.00	0.99	-0.01 (-0.01– -0.004)	0.00	1.00
Methylene chloride	0.09	0.99	0.10 (0.03–0.17)	0.15	0.98

^a^ Sensitivity/Specificity/PPV/NPV of self-reports when compared with the JEM

### Association of TGCT with occupational solvent exposure

Table 4 shows the adjusted OR for TGCT associated with selected solvents obtained with two approaches of exposure assessment. According to JEM, a positive association between TCE exposure and TGCT risk was observed (OR 1.80, 95% CI 1.12–2.90). The association seemed to be mainly carried by the high TCE exposure with an OR of 2.23 (95% CI 1.19–4.18). A positive association between TGCT risk and KetEst exposure was also observed (OR 1.63, 95% CI 1.16–2.30), mainly driven by the low-exposure category, with an OR of 1.74 (95% CI 1.10–2.74). For F&P solvents, low-exposure was associated with an OR of 1.48 (95% CI 1.06–2.07). The remaining solvents, ie, alcohols, PCE and MC disclosed OR >1 but with wide 95% CI, including 1 (alcohols: OR 1.18, 95% CI 0.88–1.59; PCE: OR 1.18, 95% CI 0.40–3.49; MC: OR 1.02, 95% CI 0.57–1.85).

**Table 4:** Odds ratios (OR) for testicular germ cell tumors (TGCT) in relation to exposure to organic solvents, using two exposure assessment methods (TESTIS study, 2015–2018). [JEM=job-exposure matrix; CI=confidence interval; Ca/Co=cases/controls].

Solvents exposure		JEM-based exposure assessment			Combined exposure assessment	
		Ca/Co	OR (95% CI) ^a^		Ca/Co	OR (95% CI) ^a^
Alcohols						
	Not exposed	309/499	1.00		300/476	1.00
	Exposed	111/151	1.18 (0.88–1.59)		120/174	1.08 (0.81–1.44)
	Low	58/76	1.35 (0.92–1.98)		54/88	1.02 (0.70–1.50)
	High	53/75	1.03 (0.69–1.54)		66/86	1.13 (0.82–1.70)
Ketones & esters						
	Not exposed	333/562	1.00		317/520	1.00
	Exposed	87/88	1.63 (1.16–2.30)		103/130	1.26 (0.92–1.73)
	Low	46/44	1.74 (1.10–2.74)		42/65	1.02 (0.66–1.59)
	High	41/44	1.52 (0.96–2.43)		61/65	1.50 (1.01–2.24)
Fuels & petroleum-based solvents						
	Not exposed	238/425	1.00		284/490	1.00
	Exposed	182/225	1.43 (1.09–1.87)		136/160	1.44 (1.08–1.92)
	Low	93/112	1.48 (1.06–2.07)		62/80	1.30 (0.88–1.90)
	High	89/113	1.38 (0.98–1.94)		74/80	1.59 (1.09–2.30)
Trichloroethylene						
	Not exposed	377/611	1.00		388/628	1.00
	Exposed	43/39	1.80 (1.12–2.90)		32/22	2.59 (1.42–4.72)
	Low	16/20	1.39 (0.70–2.77)		16/12	2.35 (1.04–5.31)
	High	27/19	2.23 (1.19–4.18)		16/10	2.87 (1.25–6.60)
Perchloroethylene						
	Not exposed	414/642	1.00		411/645	1.00
	Exposed	6/8	1.18 (0.40–3.49)		9/5	2.63 (0.83–8.27)
	Low					
	High					
Methylene chloride						
	Not exposed	397/620	1.00		411/641	1.00
	Exposed	23/30	1.02 (0.57–1.85)		9/9	1.55 (0.59–4.09)
	Low	12/16	0.89 (0.39–2.00)			
	High	11/14	1.19 (0.52–2.74)			

^a^ Estimates obtained comparing TGCT cases to group A and group B controls combined and adjusted for sibship size, being born from multiple pregnancy, personal history of testicular trauma, family history of TGCT and family history of cryptorchidism. Analysis was restricted to subjects with no missing data for the adjustment variables (N=12). If the “Low”/”High” categories have <5 cases or controls, the categories are grouped into “None” or “All” if the number of subjects was sufficient, otherwise the line is shaded.

Overall, all positive associations with TGCT observed with JEM were also observed with the JEM+SQ+EA method. The association between TGCT and TCE exposure showed a higher OR than with JEM assessment alone (OR_never/ever_ 2.59, 95% CI 1.42–4.72), and with an increasing OR with the level of exposure (OR_low-exposed_ 2.35, 95% CI 1.04–5.31 / OR_high-exposed_ 2.87, 95% CI 1.25–6.60). For KetEst, the “never/ever exposed” showed this time a lower OR (OR 1.26, 95% CI 0.92–1.73) compared to first approach. Nevertheless, a positive association with the highly exposed category was observed (OR 1.50, 95% CI 1.01–2.24). The association between TGCT risk and F&P solvents was also observed in the “never/ever exposed” category (OR 1.44, 95% CI 1.08–1.92) that seemed to be driven mainly by the high-exposed category (OR 1.59, 95% CI 1.09–2.30). Exposure to alcohols showed an OR closer to 1 (OR 1.08, 95% CI 0.81–1.44). In contrast, the OR observed with PCE exposure was higher in JEM+SQ+EA method, but with an even wider CI (OR 2.63, 95% CI 0.83–8.27). Exposure to MC showed also a higher OR (OR 1.55, 95% CI 0.59–4.09), but with an even wider CI, still including the null hypothesis.

In the sensitivity analyses, we repeated the analyses classifying jobs periods with a JEM-based P of 10–70% as unexposed. Overall, no noticeable change was observed with positive associations of similar magnitude between TGCT risk, and KetEst, and F&P solvents. For TCE, the OR for the high-exposed category was higher compared to the main analyses, although with a wider CI (OR 2.97, 95% CI 1.19–7.45) (supplementary table S7).

Moreover, results were not substantially modified in the sensitivity analyses excluding TGCT cases with personal history of cryptorchidism (supplementary table S8). However, additional adjustment for age, lead to an increase of the OR for ever exposed to PCE (OR 3.55, 95% CI 1.00–12.52), but with wider CI (supplementary table S8).

## Discussion

In this study, occupational exposure to selected solvents were positively associated with an increased TGCT risk, using two complementary exposure assessment methods, ie, the Matgéné JEM and an assessment combining JEM-, SQ-, and EA-data. Elevated OR were consistently observed with the two approaches among men ever exposed to TCE. The results suggest that this solvent might be involved in the pathogenesis of TGCT in occupationally exposed men. Moreover, exposure to KetEst and to F&P solvents was positively associated with TGCT with both approaches, but these associations seemed mainly driven by the low-exposure category in the JEM method and the high-exposure category in the JEM+SQ+EA method.

### TGCT risk and solvent exposure

The proportion of men ever exposed to any solvents reported in our study was higher than observed in the population of French workers in 1999 (22). Data from the ICARE case–control study (23) (conducted between 2001 and 2007 in France) having used the same JEM (Matgéné) to assess lifetime occupational exposure to solvents. Barul et al (23) reported a lifetime occupational exposure to TCE in controls of 8%, slightly higher than the 6% observed in TESTIS, but our data were more recent and the study population younger. In contrast, the proportion of controls exposed to PCE and MC in ICARE (0.3% and 1%, respectively) was lower compared to TESTIS (1% and 5%, respectively). The prevalence of alcohol and KetEst were also lower among controls of ICARE compared to TESTIS.

To our knowledge, the present study is the first to observe a robust association between occupational exposure to TCE and TGCT risk among human. Our results also suggest a positive association with PCE exposure, although with little precision because of the small number of exposed cases. Exposure to chlorinated solvents has declined over time due to their toxicity and environmental impact, however until recently they have been widely used in modern industries (20, 24, 25). TCE is one of the most notable chlorinated solvents and IARC has classified it as “carcinogenic to humans”. Since 2014, PCE has been classified as a “probable carcinogen for humans” (9). Among the few studies that have investigated specific TCE or PCE exposure, Silver et al (26) reported that men employed in a microelectronics and business machine manufacturing facility had excess incidence of testicular cancer. These workers were likely exposed to various chlorinated solvents, including MC, TCE and PCE (27), and the hazard ratio (HR) for the cumulative TCE exposure score was elevated, but CI were wide and included the null (the HR for five years was 1.60, 95% CI 0.71–3.59) (26). Similar findings were obtained in a Finnish study, which showed an elevated standardized incidence ratio (SIR) of testicular cancer associated with “chlorinated hydrocarbons” in the high cumulative exposure category, with a lag period of ten years but also with low precision (SIR 1.42, 95% CI 0.68–2.62) (28). Although it has been observed that the testes may be a target organ following exposure to TCE in rodents (29), the underlying mechanisms are poorly understood (30). TCE is metabolized in humans via two distinct pathways: mainly in the liver through the action of cytochromes P450 and by conjugation with glutathione (29). Cytochrome P450 (CYP)2E1 is the principal P450 involved in the formation of TCE metabolites. Identification of CYP2E1 in animals testes (31, 32) suggested that TCE might also be bioactivated in the male reproductive system, leading possibly to toxic effects (29). TCE and its metabolites were found in all seminal fluid samples from eight mechanics diagnosed with clinical infertility and exposed to TCE, suggesting associations with reproductive toxicity among humans (32). Moreover, limited data point to a hormone disruption mechanism for TCE-induced testicular tumors (24, 30). In rats, PCE may induce testicular neoplasms, but no epidemiological study has yet reported a conclusive association with testicular cancer (33).

The TESTIS study also appears to be the first to observe a positive association between KetEst and TCGT among men. Since ketones and esters are often present in mixtures and have been grouped into a single family in the Matgéné JEM. Nevertheless, previous studies have observed a higher risk of testicular cancer among workers exposed to, among others, methyl isobutyl ketone (MIBK) (34). Also, an elevated SIR of testicular cancer in the higher cumulative exposure category of “other organic solvents”, which mainly included alcohols, ketones, esters and glycol ethers (SIR 1.42, 95% CI 0.68–2.62), was observed among a cohort of active Finnish men (28). KetEst are a subfamily of oxygenated solvents, some of which have been classified as a human health concern. MIBK and isophorone were classified by IARC as “a possible carcinogen” and cyclohexanone is “not classifiable as to its carcinogenicity” since 1999 (9). The European Union has also classified MIBK and isophorone as category 2 carcinogens (“substances of concern due to possible carcinogenic effects”) and methyl butyl ketone (MBK) as a category 2 reprotoxic (35). In addition, morphological changes in the testes – including a reduction in the number of spermatocytes, spermatids, and spermatozoa – have been shown after daily administration of MIBK (at doses of 300 or 600 mg/kg for 4 months) in rats (36), but others have not made these observations (37).

We observed a positive association between F&P solvents and TGCT, although the present study cannot identify the particular F&P solvent by which the positive association is mainly driven. Epidemiological studies have shown that benzene exposure led to abnormal sperm production (38) and sperm chromosomal aberrations (39). It has also been suggested that the risk of testicular cancer among men serving in the Royal Navy is potentially related to fuels and exhaust fumes specific to aircraft maintenance (40). Among F&P solvents, the toxicity of benzene has been widely proven, and the IARC has classified it as a carcinogen (9). Nevertheless, most applications involving the use of pure benzene or benzene in mixtures have been replaced or reduced since the 1980s in France because of regulations (41). In addition, abnormal hormone levels (ie, reduced levels of follicle-stimulating hormone, luteinizing hormone and testosterone) have previously been observed among workers exposed to toluene (42).

We have previously observed increased TGCT risks among agricultural, electrical and electronics workers, and concluded in this paper that these jobs have been associated with TGCT in the literature over time (8). In a Nordic study based on the NOCCA-JEM, electrical and electronic equipment assembler occupations were assigned exposure to solvents, particularly TCE (43). In the Agricultural Health Study cohort, 8% of farmers use solvents an additive when mixing pesticides. In the same study, the farmers who applied pesticides were also more likely to use solvents to clean equipment (44).

### Exposure assessment

The main strength of this study is the use of two complementary exposure assessment methods, including a combined bringing together JEM and SQ with EA. This JEM+SQ+EA approach capitalizes on the well-known advantages of JEM, such as time savings and generally good specificity (20), while allowing for consideration of the particularities of some jobs incorporating self-reported exposure data. This combination is complemented by the expert approach still representing the gold-standard of exposure assessment, based on detailed questionnaire responses assuming to provide more accurate exposure estimates than JEM-based data (11). It also enables a faster retrospective expert assessment by using their expertise only on discordant assessments between JEM and SQ. The a priori defined decision rules facilitated the job-by-job examination by providing initial estimates of exposure that can be modulated by the experts according to the subject-reported task information (45).

It also appears that analyses based on this combined assessment showed a stronger association than analyses based on the JEM assessment alone, possibly due to reduced exposure misclassification. The use of JEM may generate non-differential misclassification and could result in an average bias toward the null (46). A review of the literature on retrospective exposure assessment methods used in occupational health risk assessment concluded that the use of a combination of different methods, including consideration of multiple tasks performed and other subject-specific data within a job, could be a key element for more reliable reconstruction of past exposures (17).

Our study had several additional strengths. Experienced industrial hygienists developed the Matgéné JEM, which provided the initial exposure estimates in this study, specifically for the French population (20). The JEM integrates the P, L semi-quantitative indices and the period of exposure, which allows the assignment of exposure to evolve over time, in connection with the evolution of techniques, occupation conditions and regulations (20).

Our study had limitations. To date, the risk factors for TGCT are not well known, so a data-driven approach was preferred to a directed acyclic graph approach to select covariates that may account for variance in the dependent variable. In addition, to avoid that there may be variables that the model might not have considered in this way, the choice of TGCT risk factors (ie, the covariates considered) was discussed in depth beforehand by a group of epidemiologists and oncologists. Using a “change in estimate” approach, currently the most popular data-driven method for selecting confounders (47), none of the covariates tested resulted in a change in OR >10% for the solvents of interest in our study (supplementary table S9, example of TCE). The number of exposed cases was small for some solvents, resulting in large CI and preventing analysis by histologic subtype or level of exposure to PCE or MC. Finally, the quality of the exposure assessment in the JEM+SQ+EA method depends on the ability of subjects to provide valid information about their occupations, which can be influenced by the duration of employment and recall period, length of the interview etc (45). With self-reported information, we cannot exclude the possibility that bias due to differential recall of occupational tasks and their characteristics by cases and controls may have been introduced into the JEM+SQ+EA method. Other limitations of the study design may be highlighted. Young men are known to be a difficult group to approach and less likely to participate in research than other population groups (48), making it difficult to determine a population-based control group representative of the general population. As previously proposed (49), we chose two distinct control groups to test our hypotheses on populations presenting different aspects of the general population. Hospital-based control prospective recruitment aimed to facilitate the recruitment of the study population to minimize non-response bias in the present study as well as to support biological sampling (50). In France, since 2010 legislation has required that all patients be informed about fertility preservation before the start of any gonadotoxic treatment (51). Thus, although not all TGCT patients in France wish to perform sperm cryopreservation, an increasing number of these procedures has been observed over the last 20 years, consistent with the TGCT incidence rates (52). Regarding controls, while we are not able to ascertain whether controls are representative of the source population from which the cases originate, recruitment of cases and controls was performed in centers with regional coverage and similar catchment populations. In addition, although we cannot exclude socio-economic factors to be associated with attending specialized fertility clinics, there were differences neither in terms of participants’ early life characteristics or socio-economic status at interview nor mothers’ socio-economic status at subjects’ birth, between group A and group B controls (18).

### Concluding remarks

This study suggests that men occupationally exposed to TCE, KetESt and F&P solvents have an increased TGCT risk. Further studies investigating this relationship are warranted. The mechanisms that might explain these associations remain to be elucidated. Finally, the combined use of the JEM and self-reported exposure information collected through SQ and complemented by EA, appears to be an appealing approach to consider in retrospective exposure assessment to improve the reliability of the characterization of specific professional exposures while considering individual variations of exposures within a same job.

## Supplementary material

Supplementary material

## Data Availability

Data are available on reasonable request. The data collection process is described in the Methods section of this article.

## Collaborators

TESTIS Study Group: Véronique Drouineaud, Céline Chalas, Aurélie Vincent, Anne-Sophie Gille, Laurianne Kremer, Myriam Virlouvet, Lucile Ferreux, Guillemette Perier, Pauline Peretout, and Diane Rivet (Cochin Hospital, Paris); Rachel Levy and Isabelle Berthaut (Tenon Hospital, Paris); Florence Eustache, Nathalie Sermonande, Marine Durand, Charlène HARBEMONT (Jean Verdier Hospital, Paris); Jeanne Perrin, Jacqueline Saias-Magnan, Carole Daoud-Deveze, and Catherine Metzler-Guillemain (La Conception Hospital, Marseille); François Isus, (Paule de Viguier Hospital,Toulouse); Florence Brugnon, Valérie Bruhat, and Cyril Bouche (Estaing Hospital, Clermont-Ferrand); Isabelle Koscinski, Cécile Greze, Françoise Schmitt, Marius Teletin, and Nathalie Guyomard (Obstetric medico-surgical centre, Strasbourg); Aline Papaxanthos, Volcy Soula, and Lucie Chansel (Pellegrin maternity Hospital, Bordeaux); Sandrine Giscard d’Estaing, Pascale Dehee, and Delphine Yalcinkaya (Femme-Mère-Enfant Hospital, Lyon); Cynthia Frapsauce, Anne Viallon and Catherine Guerin (Bretonneau Hospital, Tour); Berengere Ducrocq, Florian Dossou Gbete (Calmette Hospital, Lille); Amélie Ancelle, Emeline Bovet-Courtois(Caen Hospital, Caen); Oxana Blagosklonov, and Séverine Bey (Jean Minjoz Hospital, Besançon); Célia Ravel, Guilhem Jouve, Ségolène Veau, Laurent Vandenbroucke and Agnès Letremy (South Hospital, Rennes); Patricia Fauque, Julie Barberet, and Céline Bruno (Dijon Hospital, Dijon); Emmanuelle Thibault, Pierre Besnier and Adrien Perche (l’Archet Hospital, Nice); Catherine Diligent, and Christel Hersant (regional University maternity Hospital, Nancy); Vanessa Loup-Cabaniols, Anna Gala, and Christelle Saintpeyre (Arnaud de Villeneuve Hospital, Montpellier); Sylvianne Hennebicq, and Julien Bessonnat (Couple-Enfant Hospital, Grenoble); Marie-Claude Blocquaux(Maison Blanche Hospital, Reims); Rémi Béranger (University Hospital Rennes, Irset (Institut de Recherche en Santé, Environnement et Travail, Rennes); Helen Boyle and Aude Fléchon (Centre Léon Bérard, Lyon).
