# Supplementary material for: Occupational exposure to organic solvents and the risk of developing testicular germ cell tumors (TESTIS study): Effect of combined exposure assessment on risk estimation
Source: Scand J Work Environ Health. 2024 Jun 27;50(5):359–71. doi: 10.5271/sjweh.4161 (PMC11245321; doi:10.5271/sjweh.4161)
Supplement: Supplementary material [file SJWEH-50-359-S001.pdf]

Occupational exposure to organic solvents and the risk of developing testicular germ cell tumors (TESTIS study): Effect of combined exposure assessment on risk estimation[1]

by Margot Guth, PhD,[2] Corinne Pilorget, PhD, Marie Lefevre, MSc, Astrid Coste, PhD, Aurélie Danjou, PhD, Brigitte Dananché, DipEng, Delphine Praud, PhD, Olivia Pérol, PhD, Myriam Daudin, MD, Marie-Ange Clarotti, MD, Stéphanie Lattes, MD, Céline Bouillon, MD, Adèle Paul, MD, Joachim Schüz, PhD, Louis Bujan, MD, PhD, Ann Olsson, PhD, Béatrice Fervers, MD, PhD, Barbara Charbotel, MD, PhD, the TESTIS study group.

1. Supplementary material
2. Correspondence to: Béatrice Fervers - Prevention Cancer Environnement Departement, Centre Léon Bérard, Lyon, France. [E-mail: beatrice.fervers@lyon.unicancer.fr]

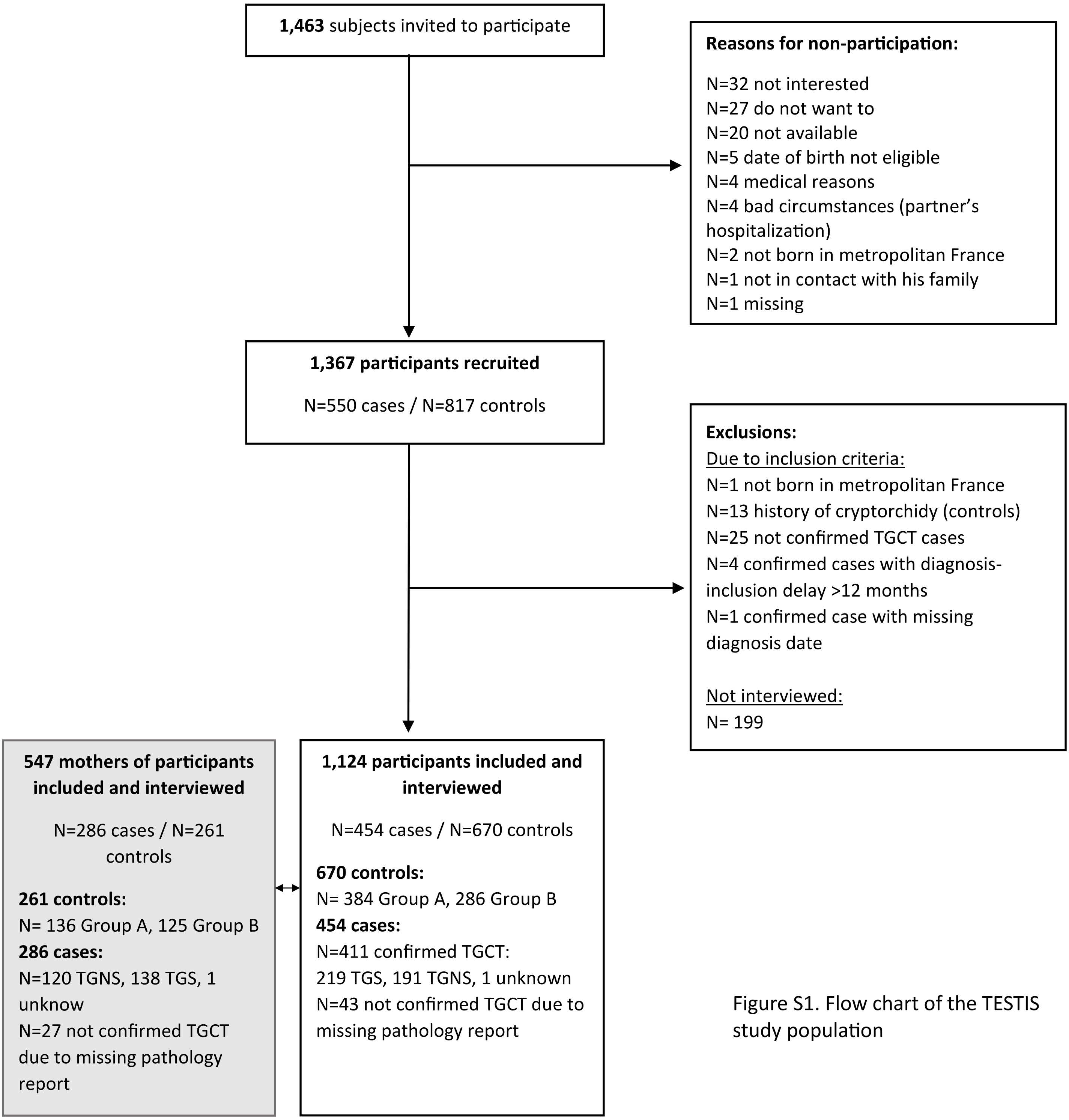

Figure S1. Flow chart of the TESTIS study population

Table S1. Job concerned by exposure to solvents reported in the exposure-specific questionnaires, TESTIS study, 2015-2018.

Self-reported occupational exposures to solvents	*Exposed*		*Don't know*		*Not exposed*	
	N (%)[1*]	*N « job-periods» (%)*[1]	*N (%)*[2*]	*N « job-periods» (%)*[2]	*N (%)*[3*]	*N « job-periods» (%)*[3]
**Alcohols**	164 (14.6)	279 (6.8)	38 (3.4)	64 (1.6)	922 (82.0)	3740 (91.6)
**Ketones and esters**	224 (19.9)	362 (8.9)	46 (4.1)	79 (1.9)	854 (76.0)	3642 (89.2)
**Fuels & petroleum-based solvents** (white spirit, kerosene, gasoline fuels …)	238 (21.2)	402 (9.8)	33 (2.9)	61 (1.5)	853 (75.9)	3620 (88.7)
**Trichloroethylene**	74 (6.6)	115 (2.8)	174 (15.5)	292 (7.2)	876 (77.9)	3676 (90.0)
**Perchloroethylene**	21 (1.9)	28 (0.7)	188 (16.7)	317 (7.8)	915 (81.4)	3738 (91.6)
**Methylene chloride**	25 (2.2)	55 (1.3)	172 (15.3)	282 (6.9)	927 (82.5)	3746 (91.7)

[1] Defined as having replied « yes » to the question "Have you ever used or been exposed through your workplace neighbors to solvents, thinners, or degreasing agents?"; [1*] Defined as having replied at least 1 time "yes" to the same question on the professional calendar.

[2] Defined as having replied « don't know » to the same question; [2*] Defined as having replied at least 1 time "don't know" and none "yes" to the same question on the professional calendar.

[3] Defined as having replied « no » to the same question; [3*] Defined as ever having replied « no » to the same question on the professional calendar.

Table S2. Example of changes over time in exposure to trichloroethylene for the same job, in the Matgéné job exposure matrix

ISCO codes	ISCO label	NAF codes	NAF label	Prob (%)	Int	Freq	Period
01420	Chemical technician	24.3Z	Paint and varnish production	[1-10 %[	[51-100] ppm	[20-30[%	1950-1969
01420	Chemical technician	24.3Z	Paint and varnish production	[10-20 %[	[51-100] ppm	[10-20[%	1970-1994
01420	Chemical technician	24.3Z	Paint and varnish production	[1-10 %[	[26-50] ppm	[20-30[%	1995-2012
01420	Chemical technician	24.3Z	Paint and varnish production	[1-10 %[	[5 – 25] ppm	[10-20[%	2013-2021

Abbreviations: Prob: Probability; Int: Intensity; Freq: Frequency

Table S3. Indices from JEMs for all solvents and weight values used for the cumulative exposure estimates calculations.

Probability of exposure	Weight [a]	Frequency of exposure	Weight [a]	Solvent	Intensity of exposure	Weight [a]	At least one solvent: Level of exposure	At least one solvent: Weight [a]
**Chlorinated solvents**								
< 1%	**0**	< 1%	**0**	**Trichloroethylene, Perchloroethylene, Methylene chloride**	< 5 ppm	**0**	not exposed	**0**
[1 - 10%[	**0.05**	[1 - 10%[	**0.05**		[5 – 25 ppm]	**15**	very low	**15**
[10 - 20%[	**0.15**	[10 - 20%[	**0.15**		[26 – 50 ppm]	**37.5**	low	**37.5**
[20 - 30%[	**0.25**	[20 - 30%[	**0.25**		[51 – 100 ppm]	**75**	medium	**75**
[30 - 39%[	**0.35**	[30 - 40%[	**0.35**		> 100 ppm	**150**	high	**150**
[40 – 50%[	**0.45**	[40 - 50%[	**0.45**	**Chloroform**	not exposed	**0**		
[50 - 60%[	**0.55**	[50 - 60%[	**0.55**		very low	**0.625**		
[60 - 70%[	**0.65**	[60 - 70%[	**0.65**		low	**1.875**		
[70 - 80%[	**0.75**	[70 - 80%[	**0.75**		medium	**3.75**		
[80 - 90%[	**0.85**	[80 – 90%[	**0.85**		high	**10**		
≥ 90%	**0.95**	≥ 90%	**0.95**	**Carbon tetrachloride**	not exposed	**0**		
					very low	**0.25**		
					low	**0.75**		
					medium	**1.5**		
					high	**3**		
**Fuels and Petroleum-based solvents**								
< 1%	**0**	< 0.5%	**0**	**Benzene**	0	**0**	not exposed	**0**
[1 - 10%[	**0.05**	[0.5 – 5%[	**0.025**		[0.1 – 1 ppm]	**0.5**	low	**3.5**
[10 - 50%[	**0.30**	[5 – 30%[	**0.175**		]1 – 5 ppm]	**3**	medium	**23**
[50 - 90%[	**0.70**	[30 – 70%[	**0.50**		]5 – 15 ppm]	**10**	high	**70**
[90 - 100%]	**0.95**	≥ 70%	**0.85**		> 15 ppm	**20**		
				**White spirits and other aromatics**	< 1 ppm	**0**		
					[1 – 20 ppm]	**10**		
					]20 – 50 ppm]	**35**		
					> 50 ppm	**65**		
				**Special petroleum products and other aliphatic**	not exposed	**0**		
					low	**10**		
					medium	**35**		
					high	**65**		
				**Gasoline**	< 1 ppm	**0**		
					[1 – 50 ppm]	**25**		
					]50 – 150 ppm]	**100**		
					> 150 ppm	**200**		
				**Kerosene/diesel oil/fuel oils**	not exposed	**0**		
					low	**25**		
					medium	**100**		
					high	**200**		
**Oxygenated solvents**								
< 1%	**0**	< 1%	**0**	**Alcohol, Ketones/esters**	not exposed	**0**	not exposed	**0**
1 - 9%	**0.05**	1 - 9%	**0.05**		very low	**1**	very low	**1**
10 - 19%	**0.15**	10 - 19%	**0.15**		low	**4**	low	**4**
20 - 29%	**0.25**	20 - 29%	**0.25**		medium	**8**	medium	**8**
30 - 39%	**0.35**	30 - 39%	**0.35**		high	**16**	high	**16**
40 - 49%	**0.45**	40 - 49%	**0.45**	**Diethyl ether, Ethylene glycol, Tetrahydrofuran**	not exposed	**0**		
50 - 59%	**0.55**	50 - 59%	**0.55**		low	**1**		
60- 69%	**0.65**	60 - 69%	**0.65**		medium	**3**		
70 - 79%	**0.75**	70 - 79%	**0.75**		high	**6**		
80 - 89%	**0.85**	80 - 89%	**0.85**					
≥ 90%	**0.95**	≥ 90%	**0.95**					

Table S4. Combined approach combing JEM and SQ assessments with expert assessment: number of jobs-periods concerned by each of the seven conditions and decision rules defined, TESTIS study, 2015-2018.

Condition No.	Alcohols	KetEst	Petroleum solvents	TCE	PCE	MC
**1**	82	111	233	22	0	8
**2**	3091	3258	2804	3581	3767	3682
**3**	7	1	19	0	0	0
	209	11	127	0	0	0
**4**	218	182	188	17	1	7
**5**	16	11	280	100	17	70
**6**	56	61	36	30	9	21
	84	103	75	37	15	22
**7**	50	75	51	26	4	3

Abbreviations – E: Exposed; Not E: Not Exposed; KetEst: Ketones and esters; Petroleum solvents: Fuels & Petroleum-based solvents; TCE: Trichloroethylene; PCE: Perchloroethylene; MC: Methylene chloride.
2,540 job-periods were concordantly assessed as exposed or unexposed by the JEM and the SQ, and 1,273 jobs-periods with at least one discordant exposure assessment between JEM and SQ, i.e., assessed by the JEM as exposed [or not exposed] while assessed by the SQ as unexposed [or as exposed].

Table S5. The cut-offs of solvent exposure, TESTIS study, 2015-2018.

	50th percentiles values of subjects' exposures **JEM assessment**	50th percentiles values of subjects' exposures **Combined assessment method**
Alcohols	8.17	7.32
Ketones and esters	7.56	5.20
Trichloroethylene	6.19	39.43
Perchloroethylene	6.50	22.8
Methylene chloride	11.25	75.53
Fuel & Petroleum-based solvents	38.94	119.7

Table S6. Prevalence of occupational exposure to organic solvents for cases and controls, TESTIS study, 2015-2018.

**Solvents**	**JEM' assessment**						**Combined assessment method**					
	Cases exposed		Controls exposed		Subjects exposed		Cases exposed		Controls exposed		Subjects exposed	
	N	%	N	%	N	%	N	%	N	%	N	%
Alcohols	114	27%	151	23%	265	24%	124	29%	175	27%	299	28%
Ketones and esters	90	21%	90	14%	180	17%	106	25%	132	20%	238	22%
Trichloroethylene	44	10%	41	6%	85	8%	32	7%	23	4%	55	5%
Perchloroethylene	6	1%	8	1%	14	1%	9	2%	5	1%	14	1%
Methylene chloride	24	6%	30	5%	54	5%	9	2%	9	1%	18	2%
Fuel & Petroleum-based solvents	188	44%	230	35%	418	39%	148	34%	179	27%	327	30%

Table S7. Sensibility analysis when "discordant" jobs evaluated by the JEM between 10 and 70% were classified as non-exposed, TESTIS study, 2015-2018.

**Solvents exposure**		**JEM' Exposure assessment**		**Combined assessment method**	
		Ca/Co	OR (95% CI) [a]	Ca/Co	OR (95% CI) [a]
Alcohols	Not exposed	309/499	1.00	346/539	1.00
	Exposed	111/151	1.18 (0.88-1.59)	74/111	0.99 (0.71-1.39)
	Low	58/76	1.30 (0.88-1.92)	42/56	1.16 (0.75-1.81)
	High	53/75	1.07 (0.72-1.60)	32/55	0.83 (0.52-1.34)
Ketones and esters	Not exposed	333/562	1.00	356/568	1.00
	Exposed	87/88	**1.63 (1.16-2.30)**	64/82	1.23 (0.85-1.79)
	Low	46/44	**1.74 (1.10-2.74)**	25/42	0.89 (0.51-1.53)
	High	41/44	1.52 (0.96-2.43)	39/40	1.61 (0.98-2.62)
Fuels & petroleum-based solvents	Not exposed	238/425	1.00	317/542	1.00
	Exposed	182/225	**1.43 (1.09-1.87)**	103/108	**1.62 (1.17-2.24)**
	Low	93/112	**1.47 (1.05-2.05)**	55/54	**1.64 (1.07-2.52)**
	High	89/113	1.39 (0.99-1.96)	48/54	**1.58 (1.03-2.45)**
Trichloroethylene	Not exposed	377/611	1.00	396/632	1.00
	Exposed	43/39	**1.80 (1.12-2.90)**	24/18	**2.37 (1.22-4.60)**
	Low	16/20	1.31 (0.65-2.65)	11/10	1.86 (0.73-4.73)
	High	27/19	**2.30 (1.23-4.30)**	13/8	**2.97 (1.19-7.45)**
Perchloroethylene	Not exposed	414/642	1.00		
	Exposed	6/8	1.18 (0.40-3.49)		
	Low				
	High				
Methylene chloride	Not exposed	397/620	1.00	415/644	1.00
	Exposed	23/30	1.02 (0.57-1.85)	5/6	1.30 (0.39-4.36)
	Low	12/16	0.89 (0.39-2.00)		
	High	11/14	1.19 (0.52-2.74)		

Abbreviations - OR: odds ratio; 95% CI: confidence interval of 95%; Ca/Co (ever)/(never):

[a] Estimates obtained comparing TGCT cases to group A and group B controls combined and adjusted for sibship size, being born from multiple pregnancy, personal history of testicular trauma, family history of TGCT and family history of cryptorchidism. Analysis was restricted to subjects with no missing data for the adjustment variables (N=12). Results presented if a job was held by more than five cases and five controls.

Table S8. Odds ratios and 95% confidence intervals for TGCT associated with occupational exposure to solvents ; use of the combined assessment method in sensitivity analyses,TESTIS study, 2015-2018.

**Solvents exposure**		**Excluding cases with personal history of cryptorchidism**		**Excluding cases not confirmed by pathology reports**		**Additional adjustment for age**	
		Ca/Co	OR (95% CI) [a,b]	Ca/Co	OR (95% CI) [a,c]	Ca/Co	OR (95% CI) [a]
Alcohols	Not exposed	279/476	1.00	267/473	1.00	272/477	1.00
	Exposed	102/174	1.01 (0.75-1.36)	112/174	1.08 (0.80-1.44)	116/174	1.11 (0.82-1.50)
	Low	47/88	0.94 (0.63-1.40)	51/88	0.98 (0.66-1.46)	54/88	1.00 (0.67-1.49)
	High	55/86	1.07 (0.73-1.57)	61/86	1.17 (0.81-1.70)	62/86	1.22 (0.84-1.80)
Ketones end esters	Not exposed	293/520	1.00	289/517	1.00	294/521	1.00
	Exposed	88/130	1.19 (0.86-1.65)	90/130	1.14 (0.82-1.57)	94/130	1.12 (0.81-1.56)
	Low	38/65	1.02 (0.65-1.60)	36/65	0.92 (0.58-1.45)	38/65	0.86 (0.54-1.37)
	High	50/65	1.37 (0.90-2.08)	54/65	1.36 (0.90-2.06)	56/65	1.39 (0.92-2.10)
Fuel & petroleum-based solvents	Not exposed	260/490	1.00	255/487	1.00	260/491	1.00
	Exposed	121/160	**1.39 (1.03-1.87)**	124/160	**1.38 (1.02-1.86)**	128/160	**1.38 (1.02-1.86)**
	Low	54/80	1.23 (0.82-1.82)	55/80	1.19 (0.80-1.77)	56/80	1.14 (0.76-1.71)
	High	67/80	**1.56 (1.07-2.27)**	69/80	**1.58 (1.08-2.30)**	72/80	**1.62 (1.11-2.38)**
Perchloroethylene	Not exposed	376/645	1.00	371/642	1.00	380/646	1.00
	Exposed	5/5	1.53 (0.42-5.59)	8/5	2.57 (0.79-8.39)	8/5	**3.55 (1.00-12.52)**
	Low						
	High						
Trichloroethylene	Not exposed	355/628	1.00	352/625	1.00	361/629	1.00
	Exposed	26/22	**2.36 (1.26-4.42)**	27/22	**2.33 (1.25-4.34)**	27/22	**2.45 (1.32-4.54)**
	Low	12/12	1.95 (0.81-4.68)	12/12	1.98 (0.83-4.73)	12/12	2.04 (0.85-4.92)
	High	14/10	**2.82 (1.20-6.64)**	15/10	**2.71 (1.16-6.33)**	15/10	**2.89 (1.24-6.74)**
Methylene chloride	Not exposed	374/641	1.00	371/638	1.00	380/642	1.00
	Exposed	7/9	1.38 (0.49-3.91)	8/9	1.50 (0.55-4.10)	8/9	1.48 (0.54-4.07)
	Low						
	High						

Abbreviations - OR: odds ratio; 95% CI: confidence interval of 95%; Ca/Co (ever)/(never):

[a] Estimates obtained comparing TGCT cases to group A and group B controls combined and adjusted for sibship size, being born from multiple pregnancy, personal history of testicular trauma, family history of TGCT and family history of cryptorchidism. Analysis was restricted to subjects with no missing data for the adjustment variables (N=12). Results presented if a job was held by more than five cases and five controls.

[b] 40 cases with personal history of cryptorchidism were excluded from the analyses. [c] 43 cases not confirmed by pathology reports, were excluded from the analyses..

Table S9. Analysis for a “change in estimate” adjustment method suggested by Talbot and Massamba, 2019 (Talbot & Massamba, 2019), example of results with individual exposure to TCE, TESTIS study.

Effect		OR	Lower 95 CI%	Upper 95 CI%	Δ OR: “change in estimate” method
expo_trichlo	"Exposed vs Not Exposed"	2.53	1.43	4.51	
birth_weight_low	"<2500 vs 2500-4000"	1.11	0.62	1.98	
	">=4000 vs 2500-4000"	1.24	0.81	1.91	
expo_trichlo	"Exposed vs Not Exposed"	**2.31**	**1.28**	**4.16**	9%
Smoking status	"Former smoker vs Never smoker"	0.97	0.71	1.33	
	"Current smoker vs Never smoker"	1.27	0.95	1.70	
expo_trichlo	"Exposed vs Not Exposed"	**2.41**	**1.35**	**4.30**	5%
Gestational age (weeks)	"<=36 vs >36"	1.42	0.85	2.38	
expo_trichlo	"Exposed vs Not Exposed"	**2.43**	**1.36**	**4.34**	4%
Born from multiple pregnancy	"Yes vs No"	2.19	1.13	4.26	
expo_trichlo	"Exposed vs Not Exposed"	**2.53**	**1.42**	**4.49**	0%
Birth order	"Second vs First"	1.08	0.82	1.43	
	"Third vs First"	1.00	0.69	1.47	
	"Fourth and more vs First"	0.45	0.24	0.84	
expo_trichlo	"Exposed vs Not Exposed"	**2.49**	**1.39**	**4.45**	2%
birth_order	"Second and more vs First"	0.97	0.76	1.25	
expo_trichlo	"Exposed vs Not Exposed"	**2.54**	**1.43**	**4.51**	0%
Sibship size	"2 vs 1"	1.89	1.11	3.23	
	"3 vs 1"	1.96	1.14	3.40	
	" >=4 vs 1"	1.41	0.80	2.50	
expo_trichlo	"Exposed vs Not Exposed"	**2.57**	**1.44**	**4.60**	1%

Table S9 *continued*. Analysis for a "change in estimate" adjustment method suggested by Talbot and Massamba, 2019 (Talbot & Massamba, 2019), example of results with individual exposure to TCE, TESTIS study.

Effect		OR	Lower 95 CI%	Upper 95 CI%	Δ OR: "change in estimate" method
Personal history of testicular trauma	" Yes vs No "	1.78	1.20	2.65	
expo_trichlo	"Exposed vs Not Exposed"	**2.42**	**1.35**	**4.32**	5%
Family history of TGCT	" Yes vs No"	3.58	1.90	6.72	
expo_trichlo	"Exposed vs Not Exposed"	**2.69**	**1.50**	**4.83**	6%
Family history of cryptorchidism	" Yes vs No"	2.20	1.18	4.10	
expo_trichlo	"Exposed vs Not Exposed"	**2.58**	**1.44**	**4.63**	2%
Geographic origin	"French by acquisition vs French by birth"	0.84	0.27	2.58	
expo_trichlo	"Exposed vs Not Exposed"	**2.55**	**1.43**	**4.54**	1%
Cannabis use at adolescence	" Yes vs No"	0.86	0.66	1.13	
expo_trichlo	"Exposed vs Not Exposed"	**2.57**	**1.45**	**4.58**	2%
Cannabis use at adolescence (frequency)	"< once / month vs Never"	0.85	0.57	1.26	
	"≥ 1 time / month vs Never"	0.62	0.35	1.10	
	"once / week vs Never"	1.31	0.80	2.15	
	"once / day vs Never"	0.77	0.47	1.24	
expo_trichlo	"Exposed vs Not Exposed"	**2.63**	**1.47**	**4.69**	4%
Cannabis use 18-25 ans	"Yes vs No "	1.09	0.85	1.41	
expo_trichlo	"Exposed vs Not Exposed"	**2.53**	**1.42**	**4.49**	0%

Table S9 *continued*. Analysis for a “change in estimate” adjustment method suggested by Talbot and Massamba, 2019 (Talbot & Massamba, 2019), example of results with individual exposure to TCE, TESTIS study.

Effect		OR	Lower 95 CI%	Upper 95 CI%	Δ OR: “change in estimate” method
Cannabis use 18-25 ans (frequency)	"< once / month vs Never"	0.87	0.60	1.26	
	"≥ 1 time / month vs Never"	0.85	0.49	1.47	
	"once / week vs Never"	1.17	0.74	1.85	
	"once / day vs Never"	1.50	1.02	2.20	
expo_trichlo	"Exposed vs Not Exposed"	**2.42**	**1.35**	**4.32**	**5%**
Alcohol consumption	"Yes vs No"	1.92	1.06	3.48	
expo_trichlo	"Exposed vs Not Exposed"	**2.65**	**1.48**	**4.72**	4%
Alcohol consumption	"< once / month vs Never"	2.23	1.20	4.15	
	"1 - 5 times / week vs Never"	1.73	0.94	3.17	
	"> 5 times / week vs Never"	2.17	0.97	4.85	
expo_trichlo	"Exposed vs Not Exposed"	**2.63**	**1.47**	**4.70**	4%
Age at voice change (years)	"< 12 vs 12-16"	0.84	0.43	1.66	
	">16 vs 12-16"	0.76	0.51	1.11	
expo_trichlo	"Exposed vs Not Exposed"	**2.64**	**1.48**	**4.71**	4%
